# Nitrite-Oxidizing Bacteria Community Composition and Diversity Are Influenced by Fertilizer Regimes, but Are Independent of the Soil Aggregate in Acidic Subtropical Red Soil

**DOI:** 10.3389/fmicb.2018.00885

**Published:** 2018-05-08

**Authors:** Shun Han, Xiang Li, Xuesong Luo, Shilin Wen, Wenli Chen, Qiaoyun Huang

**Affiliations:** ^1^State Key Laboratory of Agricultural Microbiology, Huazhong Agricultural University, Wuhan, China; ^2^Key Laboratory of Arable Land Conservation (Middle and Lower Reaches of Yangtze River), Ministry of Agriculture, College of Resources and Environment, Huazhong Agricultural University, Wuhan, China; ^3^Hengyang Red Soil Experimental Station, Chinese Academy of Agricultural Sciences, Beijing, China

**Keywords:** nitrite oxidizers, *Nitrobacter*, *Nitrospira*, fertilizer regimes, soil aggregate fractions

## Abstract

Nitrification is the two-step aerobic oxidation of ammonia to nitrate via nitrite in the nitrogen-cycle on earth. However, very limited information is available on how fertilizer regimes affect the distribution of nitrite oxidizers, which are involved in the second step of nitrification, across aggregate size classes in soil. In this study, the community compositions of nitrite oxidizers (*Nitrobacter* and *Nitrospira*) were characterized from a red soil amended with four types of fertilizer regimes over a 26-year fertilization experiment, including control without fertilizer (CK), swine manure (M), chemical fertilization (NPK), and chemical/organic combined fertilization (MNPK). Our results showed that the addition of M and NPK significantly decreased *Nitrobacter* Shannon and Chao1 index, while M and MNPK remarkably increased *Nitrospira* Shannon and Chao1 index, and NPK considerably decreased *Nitrospira* Shannon and Chao1 index, with the greatest diversity achieved in soils amended with MNPK. However, the soil aggregate fractions had no impact on that alpha-diversity of *Nitrobacter* and *Nitrospira* under the fertilizer treatment. Soil carbon, nitrogen and phosphorus in the soil had a significant correlation with *Nitrospira* Shannon and Chao1 diversity index, while total potassium only had a significant correlation with *Nitrospira* Shannon diversity index. However, all of them had no significant correlation with *Nitrobacter* Shannon and Chao1 diversity index. The resistance indices for alpha-diversity indexes (Shannon and Chao1) of *Nitrobacter* were higher than those of *Nitrospira* in response to the fertilization regimes. Manure fertilizer is important in enhancing the *Nitrospira* Shannon and Chao1 index resistance. Principal co-ordinate analysis revealed that *Nitrobacter*- and *Nitrospira*-like NOB communities under four fertilizer regimes were differentiated from each other, but soil aggregate fractions had less effect on the nitrite oxidizers community. Redundancy analysis and Mantel test indicated that soil nitrogen, carbon, phosphorus, and available potassium content were important environmental attributes that control the *Nitrobacter*- and *Nitrospira*-like NOB community structure across different fertilization treatments under aggregate levels in the red soil. In general, nitrite-oxidizing bacteria community composition and alpha-diversity are depending on fertilizer regimes, but independent of the soil aggregate.

## Introduction

Nitrification is of great importance in the nitrogen (N) cycle of agricultural ecosystems, including the oxidation processes of ammonia and nitrite. Ammonia oxidation is the first and rate-limiting step in nitrification, which is a critical aerobic process, resulting in the formation of nitrite by ammonia-oxidizing bacteria (AOB) and archaea (AOA) (Kowalchuk and Stephen, 2001; Leininger et al., 2006; Gubry-Rangin et al., 2010; Hayatsu et al., 2017), as well as some taxa called comammox belonging to the *Nitrospira* lineage II (Daims et al., 2015; van Kessel et al., 2015). Nitrite-oxidation is the second step in nitrification; catalyzed by nitrite oxidizing bacteria (NOB) which can convert nitrite to nitrate (Prosser, 1989). Compared with the study on the community structure of AOB and AOA (Kowalchuk and Stephen, 2001; Webster et al., 2005; He et al., 2007; Le Roux et al., 2008; Zhang et al., 2012), intensive investigation of the ecology of the nitrite oxidizing community has received little attention (Attard et al., 2010; Ward et al., 2011; Daims et al., 2016; Han et al., 2017).

Nitrite-oxidizers mainly comprise six bacterial genera: *Nitrobacter, Nitrospira, Nitrotoga, Nitrococcus, Nitrospina*, and *Nitrolancetus*, which are affiliated with *Alphaproteobacteria, Betaproteobacteria, Gammaproteobacteria*, and *Deltaproteobacteria*, as well as the phyla *Chloroflexi* (*Nitrolancetus*) and *Nitrospirae* (*Nitrospira defluvii*), respectively (Gould and Lees, 1960; Watson and Waterbury, 1971; Daims et al., 2001; Alawi et al., 2007; Sorokin et al., 2012). However, only *Nitrobacter*- and *Nitrospira*-like NOB are believed to play important functional roles in terrestrial ecosystems (Bartosch et al., 2002; Kim and Kim, 2006; Ke et al., 2013). *Nitrobacter*-like NOB are *r*-strategists, which prefer high substrate concentrations and have lower substrate affinity, while *Nitrospira*-like NOB are *K*-strategists with affinity for lower nitrite and oxygen concentrations (Daims et al., 2001; Blackburne et al., 2006; Nowka et al., 2015).

*Nitrobacter*-like NOB are one of the key functional players within the NOB community in tilled soils with high N availability and a high nitrite oxidation activity (Attard et al., 2010). In addition, the selection of a dominant population of *Nitrobacter* was observed under no-tillage soils. The application of sulfadiazine-contaminated pig manure decreased the diversity of *Nitrobacter*-like NOB community in the root-rhizosphere under Merzenhausen agricultural soil (Ollivier et al., 2013). In paddy pot experiment soils, *Nitrobacter*-like NOB were found to be more active in the rice rhizosphere than in the bulk soil (Ke et al., 2013). An opposite trend was also observed for *Nitrospira*-like NOB, which suggests a different habitat preference of *Nitrobacter*-like NOB and *Nitrospira*-like NOB in paddy soils. In this case, nitrite oxidation in surface soil was dominated by *Nitrospira* spp. (Ke et al., 2013). They also found that soil depth (related to O_2_ content) and sampling site (related to rice rhizosphere) affect *Nitrobacter* activities in paddy soils. Long-term straw applications selected several lineages II *Nitrospira*, which were probably affected by the increment of available soil potassium, NH_4_^+^ and NO_3_^-^, as well as decrements of available phosphorus in paddy-upland soil (Luo et al., 2017). Our previous study also showed that *Nitrospira*-like NOB community composition is more sensitive than *Nitrobacter* to land management in acid and straw application soils of a rapeseed-rice rotation field trial, and the abundance of *Nitrospira* was always greater than *Nitrobacter*; *Nitrospira* was affected by the straw application, while *Nitrobacter* was not (Han et al., 2017). It is worth mentioning that previously detected *Nitrospira* lineage II may have been comammox (Pester et al., 2014; Bertagnolli et al., 2016; Han et al., 2017). The *nxrB* from comammox *Nitrospira* does not form an independent phylogenetic clade from nitrite-oxidizing *Nitrospira* (Daims et al., 2015). Therefore, depicting the diversity and community composition of comammox *Nitrospira* using the *nxrB* gene remains a great challenge.

Soil aggregates include organic matter, minerals and microbes in the structured particles (Tisdall and Oades, 1982; Jastrow, 1996). Soil aggregates provide spatially heterogeneous habitats for a wide range of microorganisms characterized by different organic matter, predation pressure, oxygen concentrations and water contents (Six et al., 2000; Sessitsch et al., 2001; Davinic et al., 2012). Previous studies have suggested that fertilization was the major factor affecting soil AOB community structure, and aggregate fractions exhibited the secondary effect (Jiang et al., 2014). AOB community structure seemed to be more sensitive to different soil fractions and fertilizer treatments (Zhang et al., 2017). The absorption of ammonia to soil particles was found to limit its toxicity to NOB (Venterea et al., 2015). These studies suggested soil aggregates putatively shape a functional microbial community. However, little information is available regarding the effects of soil aggregation on different functional microbial communities.

To investigate the impact of both long-term fertilization treatments and soil aggregate fractions on nitrite-oxidizing bacterial community diversity, resistance and populations, a study using high-throughput sequencing of the marker genes associated with *Nitrobacter*- and *Nitrospira*-like NOB was conducted from an experimental field of red soil in Hunan, China. We aimed at identifying the main environmental factors that affect the diversity and composition of *Nitrobacter*- and *Nitrospira*-like NOB communities in this red soil. We hypothesized the following: (i) a higher influence on the NOB community putatively came from the long-term fertilization treatments than from the aggregate fraction sizes; (ii) the resistance of the NOB alpha-diversity index in response to environmental changes is both related to the strength of disturbances (associated with the fertilization regimes) and their inhabiting microenvironments (diverged by different soil particle size classes); (iii) *Nitrospira*-like NOB and *Nitrobacter*-like NOB communities were shaped by different soil properties.

## Materials and Methods

### Experimental Site and Sampling

The experimental site was located at Qiyang Red Soil Experimental Station (26°45′N, 111°52′E), Hunan Province, China. The experiment was started in 1990 with a winter wheat (*Triticum aestivum* L.) and summer maize (*Zea mays* L.) rotation system, including four treatments with three replicate plots for each treatment-type in a randomized plot design. Wheat was planted after fertilization in October and maize was planted after fertilization in April. The treatments included the following: control without fertilizers (CK, pH = 6.87); swine manure (M, pH = 6.52); chemical fertilization (nitrogen, phosphate, and potassium fertilizers, NPK, pH = 4.83); or chemical/organic combined fertilization (nitrogen, phosphate, potassium and swine manure fertilizers, MNPK, pH = 5.90). The nitrogen fertilizer was provided as urea or swine manure at 300 kg N ha^-1^, phosphate (P) as a single application of superphosphate [Ca(H_2_PO_4_)_2_] at 53 kg P ha^-1^, and potassium (K) as potassium chloride (KCl) at 100 kg K ha^-1^.

Soil samples were collected in November 2016 at a depth of 0–20 cm. Six soil cores (approximately 5 cm in diameter) were taken from each plot and mixed to form one composite sample. Samples were placed in a sterile plastic bag for transport to the laboratory within 24 h after collection. Each soil sample was divided into portions. One portion was used for DNA extraction and stored at -80°C and another portion was air-dried at room temperature for analysis of soil chemical properties.

### Soil Aggregate Fractionation Collected

Field fresh soils were gently broken apart along natural planes of weakness and then used for aggregate fractionation (Elliot, 1986). Three water-stable aggregate-size classes were manually fractionated through sieving 100 g of fresh soil on a series of three sieves (2000–250, 250–53, and <53 μm) as follows: large macroaggregates (2000–250 μm, LA), microaggregates (250–53 μm, MA) and silt+clay (<53 μm, SA). Fresh soil was processed using the wet-sieving method as follows: 50 g of soil was submerged in deionized water for 5 min at room temperature on top of a 2000 μm sieve; the sieve was manually moved up and down 3 cm, 50 times over a 2-min period. The fraction remaining on the 2000 μm sieve was collected in a plastic cup. Water plus the filtered soil was poured through a 250 μm sieve and the sieving procedure was repeated. Water plus the 250 μm fraction of soil was poured through a 53 μm sieve, and the sieving procedure repeated; the remaining 50 g of soil was repeated with the above procedure and mixed with the above obtained aggregates and then freeze-dried and weighed to determine the proportion of the entire soil weight.

### Soil Chemical Analytical Procedures and Soil DNA Extraction

Soil aggregate total carbon (TC) and total nitrogen (TN) were measured using a Vario Max element analyzer (Elementar, Hanau, Germany). Total soil phosphorus (TP) and potassium (TK) were digested by HClO_4_ and determined by molybdenum-blue colorimetry and flame photometry, respectively (Jackson, 1958). Soil total organic carbon (SOC) was measured using a potassium dichromate oxidation spectrophotometric method. The total available phosphorus (AP) was determined according to the methods described by Olsen et al. (1954). The available potassium (AK) was extracted for 30 min with 1 M NH_4_OAc (w/v, 1:10) and analyzed by atomic absorption spectrophotometry (Lanyon and Heald, 1982). Soil exchangeable ammonium (NH_4_^+^-N) contents were determined on a FIAstar 5000 Analyzer after extraction of soil aggregate with 2 M KCl (w/v, 1:5).

Soil DNA was extracted from 0.5 g samples by a bead mill homogenization procedure, using a FastDNA SPIN kit for soil and FastPrep^®^-24 (MP Biomedicals, Santa Ana, CA, United States) according to the manufacturer’s protocol. The quality and concentration of DNA were determined using a Nanodrop-2000 spectrophotometer (Nano Drop Technologies, Wilmington, DE, United States). All extracted DNA samples were then stored under -20°C for future molecular analysis.

### Measurement of *Nitrobacter*- and *Nitrospira*-Like NOB Abundance by Q-PCR

Quantitative PCR assays were conducted using an ABI7500 FAST Real-time PCR system with *nxrA* (for *Nitrobacter*-like NOB) primers (Attard et al., 2010) and *nxrB* (for *Nitrospira*-like NOB) primers (Pester et al., 2014). The 20 μl PCR reaction mixtures contained 10 μl SYBR Premix Ex Taq II (2x) (Takara Bio Inc., Shiga, Japan), 0.9 μl of a 10 mM solution of each primer, 6.2 μl of DEPC-treated water, 2.0 μl of soil extract (diluted 10-fold) or a 2.0 μl standard plasmid. A standard curve was generated using 10-fold serial dilutions of a plasmid containing a copy of the target gene. All of the qPCR was performed in triplicate and gel electrophoresis analyses were conducted to confirm the amplification specificity. To avoid inhibitory effects on qPCR, the samples were diluted 10-fold based on a pre-experiment.

### High-Throughput Sequencing and Bioinformatics Analysis

PCR amplification of *nxrA* and *nxrB* genes was performed using an Illumina MiSeq platform with the *nxrA* primers F1norA and R2norA (Attard et al., 2010) for *Nitrobacter*-like NOB and *nxrB* gene primers *nxrB*169f and *nxrB*638r (Pester et al., 2014) for *Nitrospira*-like NOB. To distinguish amplicons originating from the different soil samples, barcode oligonucleotides of 7 bp in length were ligated to each side of the purified PCR products and an equal amount of PCR products for each sample were combined in a single tube to run on the sequencing platform. Illumina reads were split based on the barcodes, which were obtained using QIIME (Caporaso et al., 2010), and the sequences of low quality (quality score < 25, length < 200 bp or 350 bp for *nxrA* and *nxrB* genes) were removed. The remaining sequences were further screened for frame shifts using the tool FrameBot from RDP’s FunGene Pipeline^1^. The remaining quality-screened sequences were clustered into operational taxonomic units (OTU) based on 95% sequence identity. In addition, values of the OTU abundance lower than 0.001% of the total sample sequence were removed and then OTU table is used for subsequent analysis.

### Statistical Analysis

Two-way analysis of variance (ANOVA) was used to analyze effects of soil aggregates and fertilization treatments on the soil variables and alpha-diversity index (Shannon and Chao1 index) and abundances by SPSS 19.0 statistical software (IBM Co., Armonk, NY, United States). Spearman’s correlation was used to determine whether the correlation between each soil variables and alpha-diversity was significant. Redundancy analysis (RDA) was carried out using the Canoco 4.5. Monte Carlo permutation test with 999 unrestricted permutations were performed to determine the extent of the environmental parameter(s) that were used to explain the nitrifier community. The two-way permutational multivariate analysis of variance (PerMANOVA) was conducted to analyze effects of the fertilization treatment and soil aggregate fractions on the nitrifier community by using PC-ORD 5.0 (Bruce McCune and MJM Software). Principal co-ordinates analysis (PCoA) was performed^2^ in the R using the “ape” package. Multi-response permutation procedures (MRPP) were performed based on the community data by using R ‘vegan’ package to test significant differences in the community composition. Aggregated boosted trees (ABT) analysis, a statistical learning method that aimed to attain both accurate prediction and explanation, was carried out using the ‘gbmplus’ package.

### Calculation of the Resistance Index

The stabilities of α-diversity index (Shannon and Chao1 index), in terms of their resistance to fertilization treatments under soil aggregate levels, were quantified by resistance indices as described by Orwin and Wardle (2004) and Delgado-Baquerizo et al. (2014) with the following equation:

$$R_{\text{s}}=1-\frac{2\times (\left|C_{0}-S_{0}\right|)}{C_{0}+(\left|C_{0}-S_{0}\right|)}$$

where *R*_S_, the value of the resistance index of alpha-diversity index (Shannon or Chao1 index) in response to a fertilization regime. *C*_0_, the value of alpha-diversity index from the control; *S*_0_, the value of alpha-diversity index from a treated plot. For example, values from the CK and fertilization treatments under soil aggregate levels were used to calculate the resistance index for the fertilization treatments. The value of the resistance index (*R*s) is bounded between -1 and 1. If the index value reaches 1, this indicates that the treatment did not cause any change in the response variable. If the index value is 0 or negative, this means that there is a 100% change or greater than 100% change in the response variable in the treatment compared with that in the control (Orwin et al., 2006).

## Results

### Soil Geochemical Factors

The results of the soil aggregate chemical properties are shown in **Table 1**. The content of soil TC and TN is the highest in macroaggregates, followed by microaggregates and silt+clay. The soil SOC, P, K, and NH_4_^+^ content was higher in macroaggregates and microaggregates and lowest in silt+clay. However, less influence on TN, TP, TK, and NH_4_^+^ across different soil aggregates was observed. Fertilizations significantly (*p* < 0.05) increased the content of all the measured soil geochemical factors, except soil TK and NH_4_^+^ content. The soil TC, TN, and SOC content are highest in the M treatment, followed by MNPK and NPK plots. The AK content was highest in the MNPK, followed by the M and NPK plots. Two-way ANOVA indicated that the fertilization treatments and soil aggregate fractions (except NH_4_^+^) had significant impacts on those measured properties (Supplementary Table S1).

**Table 1:** Soil variables in particle-size fractions under different treatments.

	Soil aggregates	Fertilizer regimes			
		CK	M	NPK	MNPK
	Macroaggregates	0.69 ± 0.09(a)(D)	2.02 ± 0.07(a)(A)	1.17 ± 0.08(a)(C)	1.71 ± 0.02(a)(B)
TC	Microaggregates	0.65 ± 0.02(a)(C)	1.79 ± 0.06(b)(A)	1.01 ± 0.04(a)(B)	1.58 ± 0.03(a)(A)
(%)	Silt+clay	0.47 ± 0.02(a)(B)	0.98 ± 0.05(c)(A)	0.74 ± 0.03(b)(A)	0.94 ± 0.02(b)(A)
	Macroaggregates	0.11 ± 0.003(a)(D)	0.23 ± 0.005(a)(A)	0.15 ± 0.008(a)(C)	0.20 ± 0.001(a)(B)
TN	Microaggregates	0.11 ± 0.001(a)(D)	0.22 ± 0.002(a)(A)	0.14 ± 0.006(a)(C)	0.19 ± 0.002(a)(B)
(%)	Silt+clay	0.10 ± 0.005(a)(B)	0.15 ± 0.003(b)(A)	0.13 ± 0.003(a)(A)	0.15 ± 0.001(b)(A)
	Macroaggregates	18.80 ± 2.90(a)(C)	38.99 ± 0.99(a)(A)	27.39 ± 1.56(a)(B)	35.00 ± 3.03(a)(A)
SOC	Microaggregates	17.78 ± 1.51(a)(C)	35.80 ± 0.39(a)(A)	23.67 ± 0.48(a)(B)	39.22 ± 5.30(a)(A)
(g/kg)	Silt+clay	14.77 ± 1.14(a)(C)	21.78 ± 1.10(b)(B)	19.72 ± 1.99(b)(B)	23.97 ± 3.93(b)(A)
	Macroaggregates	0.47 ± 0.02(a)(C)	1.83 ± 0.19(a)(A)	1.06 ± 0.05(a)(B)	1.75 ± 0.37(ab)(A)
TP	Microaggregates	0.44 ± 0.03(a)(C)	1.89 ± 0.33(a)(A)	1.00 ± 0.12(a)(B)	2.06 ± 0.30(a)(A)
(g/kg)	Silt+clay	0.44 ± 0.06(a)(C)	1.40 ± 0.19(a)(A)	0.89 ± 0.11(a)(B)	1.35 ± 0.09(b)(A)
	Macroaggregates	8.62 ± 1.35(a)(A)	7.54 ± 1.13(a)(A)	9.84 ± 2.07(a)(A)	9.84 ± 0.62(a)(A)
TK	Microaggregates	8.90 ± 0.71(a)(A)	7.75 ± 0.54(a)(A)	9.23 ± 0.95(a)(A)	8.96 ± 0.69(b)(A)
(g/kg)	Silt+clay	7.74 ± 0.53(a)(A)	6.45 ± 0.42(a)(A)	8.55 ± 0.35(a)(A)	7.14 ± 0.39(b)(A)
	Macroaggregates	5.90 ± 2.11(b)(C)	278.66 ± 29.9(a)(A)	78.93 ± 11.0(a)(B)	221.48 ± 85.9(a)(A)
AP	Microaggregates	13.41 ± 2.20(a)(C)	220.66 ± 19.0(b)(A)	80.60 ± 16.4(a)(B)	202.03 ± 34.8(a)(A)
(g/kg)	Silt+clay	7.32 ± 4.17(b)(C)	90.71 ± 7.59(c)(A)	35.86 ± 9.92(b)(B)	120.80 ± 22.6(b)(A)
	Macroaggregates	0.51 ± 0.06(a)(D)	0.94 ± 0.06(a)(C)	1.05 ± 0.09(a)(B)	1.25 ± 0.12(a)(A)
AK	Microaggregates	0.45 ± 0.02(a)(B)	0.91 ± 0.06(a)(A)	0.98 ± 0.06(a)(A)	1.00 ± 0.09(b)(A)
(g/kg)	Silt+clay	0.40 ± 0.02(a)(C)	0.69 ± 0.06(b)(B)	0.98 ± 0.04(a)(A)	0.85 ± 0.04(c)(AB)
	Macroaggregates	16.10 ± 7.22(a)(A)	15.78 ± 2.71(a)(A)	11.29 ± 1.33(a)(A)	17.21 ± 0.75(a)(A)
NH_4_^+^	Microaggregates	14.26 ± 4.86(a)(A)	17.02 ± 3.36(a)(A)	10.63 ± 0.99(a)(A)	13.95 ± 0.61(a)(A)
(g/kg)	Silt+clay	12.11 ± 1.21(a)(A)	14.60 ± 0.98(a)(A)	10.30 ± 0.84(a)(A)	12.12 ± 3.23(a)(A)

*TC, total carbon; TN, total nitrogen; TP, total phosphorus; TK, total potassium; SOC, soil organic carbon; AP, available phosphorus; AK, available potassium; NH_*4*_^*+*^-N, ammonium nitrogen.*

*Error bars represent standard error (n = 3) and are followed by a lowercase letter indicating a significant difference among three factions within each fertilizer and the capital letter indicating a significant difference among four fertilization treatments under the same soil aggregates size according to Tukey’s test (p < 0.05).*

### Abundances of *Nitrobacter-* and *Nitrospira-*Like NOB

**FIGURE 1:**
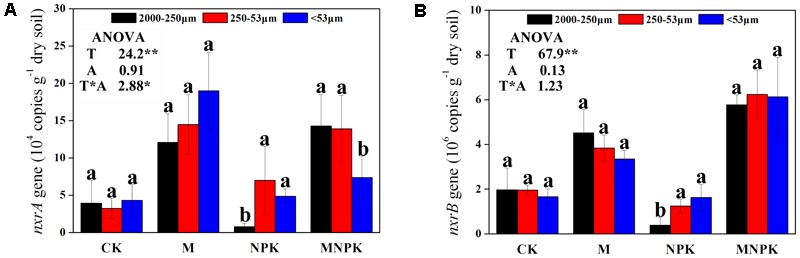
Abundances of *Nitrobacter*- **(A)** and *Nitrospira*-like NOB **(B)** of three soil fractions under different fertilizer treatments. Error bars represent standard error (*n* = 3) and are followed by a lowercase letter indicating a significant difference among three factions within each fertilizer according to the Tukey test (*p* < 0.05). T, fertilization treatment; A, soil aggregate size. ^∗^, indicate a significant difference at *p* < 0.05, ^∗∗^, indicate a significant difference at *p* < 0.01.

As shown in **Figure 1**, the abundances of the *Nitrobacter*- and *Nitrospira*-like NOB, represented by the copy numbers of the *nxrA* gene and *nxrB* gene, ranged from 0.78 ± 0.42 × 10^4^ to 19.0 ± 5.2 × 10^4^ g^-1^ dry soil, and from 0.40 ± 0.24 × 10^6^ to 6.2 ± 1.1 × 10^4^ g^-1^ dry soil, respectively. The *Nitrobacter* and *Nitrospira* abundance was significantly (*p* < 0.05) higher in the M and MNPK treatment than CK (**Figures 1A,B**), indicated that manure fertilizer could promote the abundances of NOB. No significant difference of *Nitrobacter* and *Nitrospira* abundance was observed among various soil aggregates sizes in CK and M plot. Two-way ANOVA data indicated that the *Nitrobacter* and *Nitrospira* abundance was significantly affected by fertilization treatments (*F* = 24.2, *F* = 67.9, respectively, *p* < 0.01).

### Alpha-Diversity Index of *Nitrobacter*- and *Nitrospira*-Like NOB

The community structures of *Nitrobacter*- and *Nitrospira*-like NOB were characterized by sequencing analysis of the *nxrA* and *nxrB* genes, respectively. Sequencing yielded an average of 26635 and 15812 high-quality sequences per sample for *nxrA* and *nxrB*, respectively. The constructed unique operational taxonomic units (OTUs, based on a 95% cut off) dataset was utilized in the following analysis after being normalized.

**FIGURE 2:**
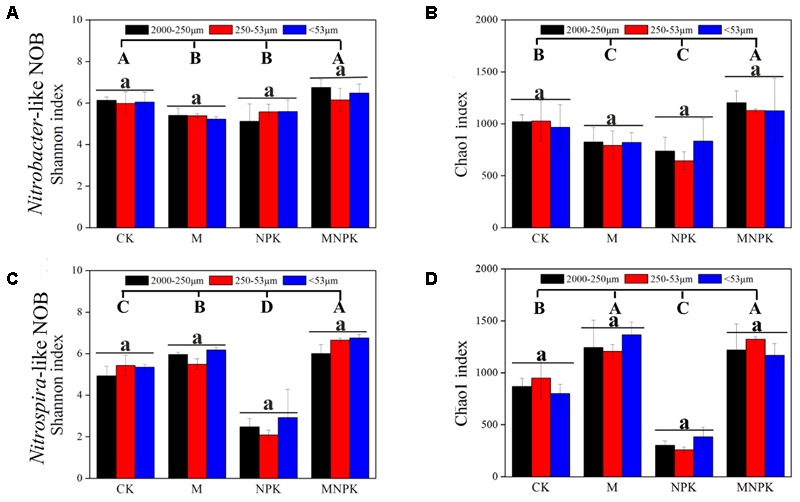
The Shannon and Chao1 diversity index of *Nitrobacter* **(A,B)** and *Nitrospira* **(C,D)**. Error bars represent standard error (*n* = 3) and are followed by a lowercase letter indicating a significant difference among three factions within each fertilizer and the capital letter indicating a significant difference among four fertilization treatments under a same soil aggregates size according to Tukey’s test (*p* < 0.05).

For *Nitrobacter*-like NOB, the Shannon index was remarkably decreased in M and NPK treatment plots and increased in MNPK compared to the CK plots (**Figure 2A**). In addition, this index was similar in different size of soil aggregate fractions under the same fertilization. For *Nitrospira*-like NOB, the Shannon index was significantly increased in M and MNPK treatments, but decreased in the NPK plot (**Figure 2C**). The index was also similar in different sizes of soil aggregate fractions under the fertilization. The trend of Chao1 index is similar to that of Shannon index for *Nitrobacter* and *Nitrospira* (**Figures 2B,D**). Two-way ANOVA showed that fertilization treatments have a strong impact (*p* < 0.01) on the Shannon and Chao1 indexes of *Nitrobacter*- and *Nitrospira*-like NOB, but soil aggregate sizes do not (Supplementary Table S1). Spearman’s rank coefficient correlation analysis showed that soil carbon, nitrogen and phosphorus in the soil had a significant correlation with *Nitrospira* Shannon and Chao1 diversity index, while total potassium only had a significant correlation with *Nitrospira* Shannon diversity index (Supplementary Table S2). However, this correlation was not detected in the *Nitrobacter*-like NOB.

**FIGURE 3:**
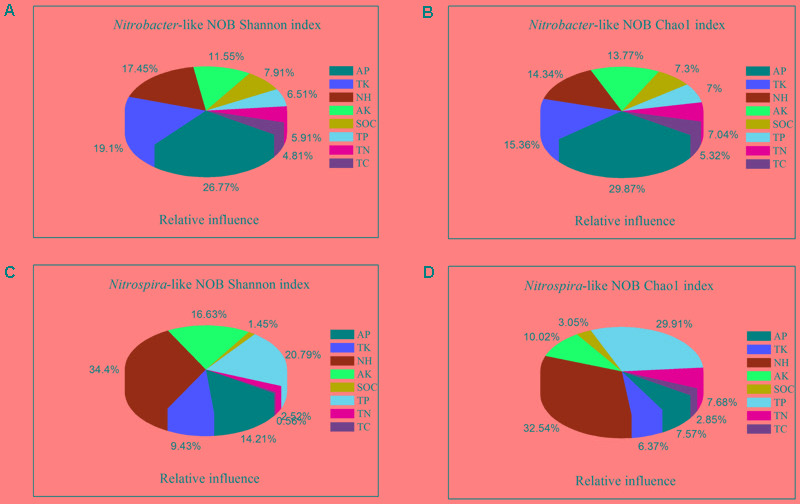
Relative influence of the driving factors for the Shannon and Chao1 diversity index of *Nitrobacter* **(A,B)** and *Nitrospira* **(C,D)** across fertilization and soil aggregate fractions by aggregated boosted trees (ABT) analysis. TC, total carbon; TN, total nitrogen; TP, total phosphorus; TK, total potassium; SOC, soil organic carbon; AP, available phosphorus; AK, available potassium; NH_4_^+^-N, ammonium nitrogen.

Aggregated boosted trees analysis indicated that soil variables were scaled differently to the alpha-diversity (Shannon and Chao1 index) of *Nitrobacter*- and *Nitrospira*-like NOB (**Figure 3**). In particular, soil AP, TK, NH_4_^+^, and AK were the most important driving factors for *Nitrobacter*-like NOB Shannon and Chao1 diversity index (**Figures 3A,B**). However, soil NH_4_^+^, TP, AK, and AP have shown a strong effect on *Nitrospira*-like NOB Shannon and Chao1 diversity index (**Figures 3C,D**). Furthermore, soil NH_4_^+^ content independently accounted for more variance in *Nitrospira* (34.4%) than in *Nitrobacter* (17.4%) in both of diversity indexes; conversely, the explanatory variables of soil AP to *Nitrobacter* (26.7%) were greater than *Nitrospira* (14.2%).

### Resistance of Alpha-Diversity of *Nitrobacter*- and *Nitrospira*-Like NOB

**FIGURE 4:**
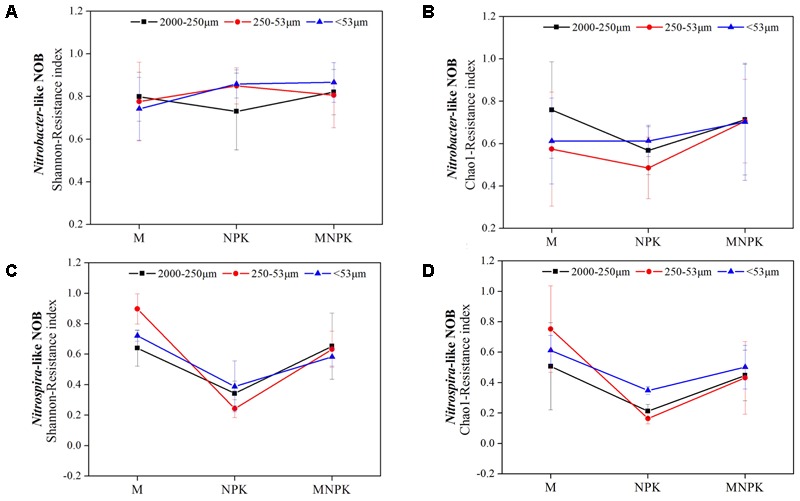
The resistance indices of *Nitrobacter* **(A,B)** and *Nitrospira* **(C,D)** Shannon and Chao1 diversity index. Error bars represent standard error (*n* = 3).

To monitor the resistance of alpha-diversity of nitrifiers, the resistance of the Shannon and Chao1 index was calculated at three fertilization treatments under the soil aggregate levels (**Figure 4**). A higher resistance (*R*_s_ > 0.7) of Shannon index was observed in *Nitrobacter*-like NOB (**Figure 4A**). However, different fertilization treatments and soil aggregate fractions have less effect on both of Shannon and Chao1 index resistance (**Figures 4A,B**). For *Nitrospira*-like NOB Shannon index resistance, the M plot has a higher resistance, followed by MNPK, and NPK has a significant (*p* < 0.05) and the lowest resistance compared to that of the M and MNPK treatments (**Figure 4C**); this outcome means that additional M and MNPK fertilizer has the capability of improving the resistance of *Nitrospira*. The trend of Chao1 index resistance of *Nitrospira* is similar to that of Shannon index (**Figure 4D**). In addition, soil aggregate sizes also have a small impact on both of Shannon and Chao1 index resistance of *Nitrospira*. Spearman’s rank coefficient correlation analysis showed that the Shannon index resistance of *Nitrospira*-like NOB was significantly associated with soil TN (*p* < 0.01), TP (*p* < 0.01), AK (*p* < 0.01), and NH_4_^+^ (*p* < 0.01) content, but this correlation was not detected in the *Nitrobacter*-like NOB (Supplementary Table S2).

### Composition of *Nitrobacter*- and *Nitrospira*-Like NOB Community

**FIGURE 5:**
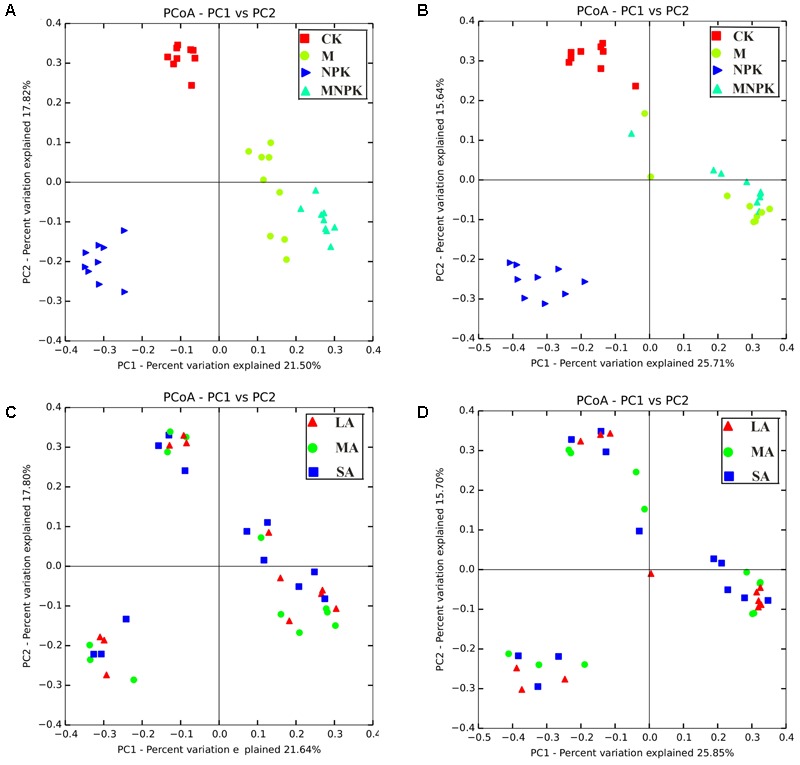
Principal co-ordinates analysis (PCoA) based on the unweighted unifrac distance showing the changes in *Nitrobacter* **(A,C)** and *Nitrospira* **(B,D)** community composition. LA, large macroaggregates (2000–250 μm), MA, microaggregates (250–53 μm), SA, silt+clay (<53 μm).

Principal co-ordinates analysis revealed that the *Nitrobacter*-like NOB community under four fertilizer regimes was well differentiated from each other along axis 1 (**Figure 5**). The *Nitrobacter*-like NOB community of CK was well separated from those of M, NPK, and MNPK along axis 2 (**Figure 5A**). In addition, a similar trend was also observed for the *Nitrospira*-like NOB community (**Figure 5B**). However, the soil aggregate fractions had no effect on the *Nitrobacter*- and *Nitrospira*-like NOB community composition (**Figures 5C,D**). MRPP analysis showed that the compositions of both *Nitrobacter*- and *Nitrospira*-like NOB community were distinctly different (*p* < 0.01) among fertilizer treatments across all soil aggregate sizes classes (Supplementary Table S3). However, significant differences among various soil aggregate classes were not detectable in all types of the fertilizer plots. Those results suggested that the long-term fertilization regimes were the main drivers in regulating NOB community structure. PerMANOVA results also indicated that both of *Nitrobacter*- and *Nitrospira*-like NOB community structure were significantly (*p* < 0.001) altered by soil fertilization treatments, and soil aggregate fractions exhibits less effects (Supplementary Table S4). Those analyses revealed that *Nitrobacter*- and *Nitrospira*-like NOB community structures were closely associated with fertilization treatments rather than soil aggregate fractions.

### The Relationship of Soil Geochemical Factors and the Composition of *Nitrobacter*- and *Nitrospira*-Like NOB Community

**FIGURE 6:**
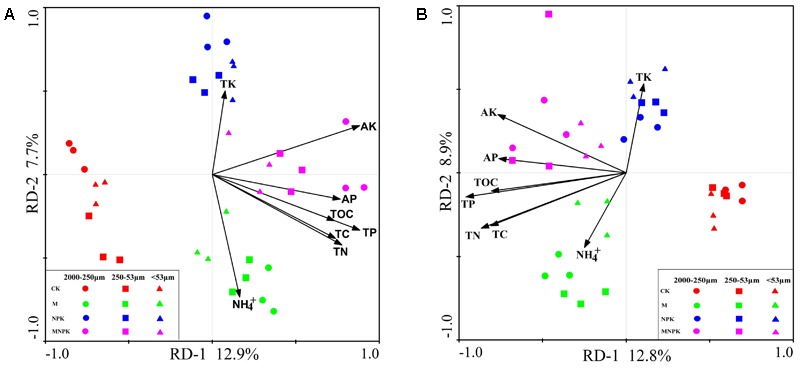
Redundancy analyses (RDA) of the correlations between soil properties and microbial *Nitrobacter* **(A)** and *Nitrospira* **(B)** community composition. TC, total carbon; TN, total nitrogen; TP, total phosphorus; TK, total potassium; SOC, soil organic carbon; AP, available phosphorus; AK, available potassium; NH_4_^+^-N, ammonium nitrogen.

Both RDA analysis and Mantel test were performed to show the relationship between *Nitrobacter*- and *Nitrospira*-like NOB community structure and the environmental factors (**Figure 6** and **Table 2**). The Monte Carlo test and Mantel results indicated that soil TC (*p* < 0.01), TN (*p* < 0.01), TP (*p* < 0.01), AP (*p* < 0.01), AK (*p* < 0.01), SOC (*p* < 0.01), and NH_4_^+^ (*p* < 0.01) content were important environmental attributes that control the *Nitrobacter*- and *Nitrospira*-like NOB community structure across different fertilization treatments in the red soil (**Table 2** and Supplementary Table S5). RDA first two components explained approximately 21% of the total variation in both of the *Nitrobacter*- and *Nitrospira*-like NOB communities (**Figures 6A,B**). The soil *Nitrobacter*- and *Nitrospira*-like NOB communities formed distinct clusters by fertilization treatments on the RDA plots, respectively. In addition, the soil *Nitrobacter*- and *Nitrospira*-like NOB community was weakly grouped into clusters by soil aggregate fractions under the same fertilization treatments.

**Table 2:** Mantel test results to discern correlation between the composition of *Nitrobacter*- and *Nitrospira*-like NOB community and soil geochemical variables.

	*Nitrobacter*-like NOB		*Nitrospira*-like NOB	
	*R*-value	*p*-value	*R*-value	*p*-value
TC	0.35	<0.001	0.32	<0.001
TN	0.43	<0.001	0.40	<0.001
SOC	0.33	<0.001	0.28	<0.001
TP	0.55	<0.001	0.53	<0.001
TK	0.06	0.165	0.04	0.433
AP	0.35	<0.001	0.37	<0.001
AK	0.38	<0.001	0.39	<0.001
NH_4_^+^	0.26	<0.001	0.34	<0.001
All factors	0.35	<0.001	0.30	<0.001

## Discussion

Different soil aggregate fractions represent different physical and chemical properties, which reflect the differentials of environmental factors and maintenance of distinct microbial assemblages within aggregate fraction (Davinic et al., 2012; Tiemann et al., 2015). Study on the soil aggregate levels potentially gives us a different point of view because different fractions represent different nutrient pools, which reflect the differentials of environmental factors. The main focus of this study was to construct a linkage between the *Nitrobacter*- and *Nitrospira*-like NOB community, soil aggregate fractions and fertilization treatments. Understanding these relationships further could be crucial for nitrogen biogeochemical cycling and sustainable agriculture.

### Responses of Diversity of *Nitrobacter*- and *Nitrospira*-Like NOB to Fertilization Treatments and Soil Aggregates

Estimating the species diversity of microorganisms is of great importance in predicting, maintaining and managing microbial communities (Hubbell, 2001). We have highlighted the significant effect of environmental variation and spatial variation on the estimation of soil nitrite-oxidizing bacterial species diversity (**Figure 2**). Our results showed that, among soil aggregate sizes, the diversity of *Nitrobacter*- and *Nitrospira*-like NOB varies to a lesser extent, while the diversity among fertilizer regimes were clearly different from the CK plot. For *Nitrobacter*, the diversity was highest in the CK and MNPK treatments, with similar values in the M and NPK treatment, which indicated that M and NPK have inhibitory effects on the diversity under aggregate levels. In particular, mixed fertilizer (chemical and organic) may recovery microbial diversity for unknown reasons. In contrast, for *Nitrospira*, the diversity was highest in the MNPK plot, followed by M and CK, and NPK treatments was the lowest. The use of organic fertilizer may offset the loss of diversity caused by chemical fertilizers. Thus, we revealed that the addition of organic fertilizer had a greater positive impact on *Nitrospira* diversity and can result in increased *Nitrospira* diversity compared to chemical fertilization under aggregate levels. In addition, genomic data showed that comammox *Nitrospira* spp. potently assimilate short-chain amides (Palomo et al., 2016). It is reasonable to hypothesize that the abundance of some comammox *Nitrospira* spp. probably increased in the soil particles from the M and MNPK fertilizer plots. However, whether manure fertilizer can really enrich comammox in soil remains an opening question.

Freitag et al. (2005) has reported the first evidence for the influence of agricultural N management regimes on the diversity of nitrite-oxidizing bacteria but failed to note the NOBs diversity regulated by which environmental factors. In this study, ABT analysis was used to better understand how the diversity could be explained by soil variables and the specific contribution of each soil variable. Soil AP, K, and NH_4_^+^ were the most important driving factors for *Nitrobacter*-like NOB community diversity. However, soil NH_4_^+^, P, and AK have shown a strong effect on *Nitrospira*-like NOB. Furthermore, ABT analysis also indicated that the alpha-diversity of *Nitrospira* was more sensitive to the nitrogen variables than *Nitrobacter* (**Figure 3**). In contrast, the alpha-diversity of *Nitrobacter* was more sensitive to the phosphorus variables than *Nitrospira.* The distinct responses of the alpha-diversity of *Nitrobacter*- and *Nitrospira*-like NOB to shift in soil factors suggests different mechanisms in controlling the composition of *Nitrobacter*- and *Nitrospira*-like NOB, which are subordinate to different phylogenetic classification and life strategies.

### Resistance of *Nitrobacter*- and *Nitrospira*-Like NOB to Fertilizer Regimes

The responses of target variables to disturbances can be quantified by a resistance index to evaluate the amount of change in response to variables caused by a disturbance as proposed by Orwin and Wardle (2004). Upon closer inspection, the alpha-diversity of *Nitrobacter* was more resistant to fertilizer regimes compared with the *Nitrospira* but was not under soil aggregate level (**Figure 4**). This may have attributed to the stronger environmental adaptability and higher resource utilization efficiency of the *Nitrobacter* community, as indicated by the lack of correlations among the diversity of *Nitrobacter*, the resistance index of *Nitrobacter* and environmental factors (Supplementary Table S2). In contrast, *Nitrospira* diversity was more disturbed in different fertilizer systems. However, manure fertilizer plays an important role enhancing *Nitrospira* diversity resistance. The distinct environmental preferences between *Nitrobacter* and *Nitrospira* may result in different responses of these functional microbial groups to fertilization treatments. We argue that knowledge on what controls soil *Nitrobacter*- and *Nitrospira*-like NOB community stability is pivotal for predicting the impacts of fertilization treatments and soil aggregate fractions on soil nitrifies community and the nitrification processes that they drive.

### Relationship Between Environmental Factors and Nitrite Oxidizer Community Structure

Organic and chemical fertilization, common agricultural practice, can significantly influence the soil aggregate size distribution, soil properties and the composition of the microbial community in the soil (Aoyama et al., 1999; Kong et al., 2005; Zhang et al., 2014, 2015). Due to the spatial and physico-chemical aspects of heterogeneous soil aggregates, soil microbes have different distribution patterns at the aggregate level (Zhang et al., 2014). Microbial community shifts were also observed with fertilizer management across soil particle-size fractions (Mummey and Stahl, 2004; Davinic et al., 2012; Lazcano et al., 2013; Zhang et al., 2017).

In this study, we found physico-chemical heterogeneity between different sizes of soil aggregates (**Table 1**). The characterization of the NOB community structure in soil aggregate level was well clustered together according to fertilization types (**Figure 5**), which indicated that fertilization treatments rather than soil aggregate sizes influence the NOB community structure in this red soil. We also observed that the fertilizer regime is a driving force of NOB community compositions. Distinctly differences in NOB community were found among fertilizer plots across the variation of soil aggregate sizes (Supplementary Table S3). Several different types of fertilizer (chemical and organic) have been added to soils to increase its nutrient content, and particularly to improve soil microenvironment fertility. Under the condition of applying organic fertilizer (M and MNPK plots), fungi can convert various organic materials into bioavailable forms in a soil ecosystem (Hoorman, 2011). It is possible that some NOB tended to use the simple organic carbon from the organic fertilizer or the organic products decomposed by fungi. Previous studies have shown that facultative/mixotrophic NOB were found to assimilate a narrow range of simple organic carbon compounds (e.g., acetate, D-lactate, pyruvate and glycerol) (Bock, 1976; Starkenburg et al., 2008). Fertilizer-induced shifts in nitrifier community composition may be explained by the direct/indirect effects of selective feeding on certain nutrient substance types as a result of the general effect of fertilizer on environmental conditions. The RDA and Mantel test indicated that soil SOC content (*p* < 0.01) was the important contributor to nitrifier community variation, which can partially support this explanation (**Figure 6** and **Table 2**). Thus, we observed a greater shift in *Nitrobacter*- and *Nitrospira*-like NOB community composition in soils amended with organic materials than those only treated with chemical fertilizers and no fertilizers.

A striking result of the present study is that we found significant relationships between all the measured soil environmental factors (except TK content) and nitrifier community compositions. Interestingly, the effects of soil variables, which were significantly (*p* < 0.01) enhanced by fertilization (except NH_4_^+^ content), on the *Nitrobacter*- and *Nitrospira*-like NOB community responded in a uniform way (**Figure 6** and **Table 2**). However, these measured environmental factors have low explanatory variables (20.6 and 21.7%, respectively) for *Nitrobacter*- and *Nitrospira*-like NOB communities; this outcome suggests that other uncharacterized biotic and abiotic factors affect the community structure of *Nitrobacter* and *Nitrospira* in soil aggregate size fractions, as one would expect for complicated microenvironment in red soil. Maixner et al. (2006) have shown that the nitrite concentration influences the structure of a *Nitrospira*-like bacterial community; however, soil aggregate nitrite is leached well-below the detection limit. Water content and oxygen availability, which are necessary for the nitrite oxidizers activity, are also the probable environmental factors that impact the nitrite-oxidizer community structure (Van Cleemput and Samater, 1996; Liu and Wang, 2013). Han et al. (2017) showed that the *Nitrospira* community changes were significantly shaped by the soil pH, whereas that of the *Nitrobacter* community was not in a rapeseed-rice rotation field trial. Also, distinct differences in *Nitrospira* community were found between the straw fertilizer and no straw. However, no obvious differences in the *Nitrobacter* were detectable. Because the water-stable soil aggregates are separated by wet-sieving method, the soil aggregate pH was not able to be accurately measured and thus not determined in this study. Therefore, in addition to the abiotic factors, the synergistic or competitive relationship between nitrite oxidizers and other microorganisms (e.g., anaerobic ammonium oxidizing bacteria) would also contribute to modulating the NOB community structure.

## Conclusion

We examined the effect of fertilizer regimes and soil aggregate fractions on soil nitrite oxidizers microbial community structure, diversity and resistance by focusing on functional genes. High throughput sequencing data indicated that the addition of M and NPK significantly decreased *Nitrobacter* diversity, while M and MNPK remarkably increased *Nitrospira* diversity, and NPK considerably decreased *Nitrospira* diversity, with the greatest diversity achieved in soils amended with MNPK. However, the soil aggregate fractions had no impact on the diversity of *Nitrobacter* and *Nitrospira* under the fertilizer treatment. Moreover, changes in diversity were largely attributed to soil phosphorus, potassium and NH_4_^+^ content. Fertilization treatments have more and less effect on the *Nitrobacter*- and *Nitrospira*-like NOB community diversity resistance index, respectively, but not soil aggregate fractions. Importantly, fertilizer regimes had significant influences on the composition and structure of soil nitrite oxidizers community; conversely, soil aggregate fractions had less effect. In addition, soil *Nitrobacter*- and *Nitrospira*-like NOB community structure were driven by nitrogen, carbon, phosphorus and available potassium content across different fertilization treatments under aggregate levels in the red soil. This work represents an important step forward in understanding nitrite oxidizers in soil aggregate levels under long-term fertilization.

## Author Contributions

WC and QH designed the study. SH and XiL conducted the experiment and analyzed the data. WC and SW collected the soil samples. SW conducted the field experiments. SH and XuL wrote the initial draft of the manuscript. WC, QH, SH, XiL, XuL, and SW contributed to revising the manuscript.

## Conflict of Interest Statement

The authors declare that the research was conducted in the absence of any commercial or financial relationships that could be construed as a potential conflict of interest.

## Footnotes

1 http://fungene.cme.msu.edu/FunGenePipeline

2 http://www.r-project.org/
