# Supplementary material for: Nitrite-Oxidizing Bacteria Community Composition and Diversity Are Influenced by Fertilizer Regimes, but Are Independent of the Soil Aggregate in Acidic Subtropical Red Soil
Source: Front Microbiol. 2018 May 8;9:885. doi: 10.3389/fmicb.2018.00885 (PMC5951965; doi:10.3389/fmicb.2018.00885)
Supplement: Supplementary file 1 [file Data_Sheet_1.PDF]

Table S1 Two-way ANOVA of soil chemical properties and Shannon and Chao1 index in soil aggregates size, fertilizer treatments. The data in bold indicated the effect was significant. *, indicate a significant difference at p< 0.05; **, indicate a significant difference at p < 0.01.

	TC	TN	SOC	TP	TK	AP	AK	NH_4^+	*Nitrobacter* Shannon	*Nitrobacter* Chao1	*Nitrospira* Shannon	*Nitrospira* Chao1
**F**	**106****	**188****	**82****	**80****	**6.8***	**223****	**137****	**5.5***	**12.1****	**23.8****	**172****	**150****
**S**	**75****	**67****	**59****	**9****	**8.5****	**118****	**26****	2.9	0.1	2.2	0.4	2.1
**S* F**	**6.7****	**10***	**7.0***	**2.3***	0.7	**53****	**4.6***	0.8	0.8	0.4	1.8	**8.9****

Notes: F, Fertilization; S, Soil aggregates size; S*F, Soil aggregates size*Fertilization.

Table S2 Spearman's rank coefficient correlation of the soil physicochemical properties and the alpha-diversity index and resistance index

	Nitrobacter-like NOB				*Nitrospira*-like NOB			
	Shannon index	Shannon Resistance index	Chao1 index	Chao1 Resistance index	Shannon index	Shannon Resistance index	Chao1 index	Chao1 Resistance index
TC	-0.14	-0.11	-0.01	0.13	**0.33****	0.37	**0.48****	**0.45***
TN	-0.18	-0.08	0.02	0.19	**0.45****	**0.54****	**0.58****	**0.49***
SOC	-0.12	-0.08	0.04	0.14	**0.35****	-0.31	**0.46***	0.32
TP	-0.04	0.03	0.16	0.24	**0.55****	**0.59****	**0.64****	0.28
TK	0.24	-0.05	0.06	0.03	**0.45****	-0.28	-0.28	-0.14
AP	0.05	0.04	0.20	0.26	**0.51****	0.08	**0.46***	-0.03
AK	-0.09	-0.06	-0.08	0.03	-0.06	**-0.39***	0.03	0.16
NH_4^+	0.06	0.05	0.17	0.17	**0.45****	**0.61****	**0.59****	0.28

Table S3 MRPP A-values and p-values of the nitrifiers community composition.

**Section of *Nitrobacter*-like NOB**				
NxrA	**Treatments**			
**Aggregate size**	CK	M	NPK	MNPK
LA vs MA	ns	ns	ns	ns
LA vs SA	ns	ns	ns	ns
MA vs SA	ns	ns	ns	ns
	**Aggregate sizes**			
**Treatment**	LA	MA	SA	
CK vs M	**0.57**(p<0.01)	**0.45**(p<0.01)	**0.35**(p<0.01)	
CK vs NPK	**0.51**(p<0.01)	**0.49**(p<0.01)	**0.32**(p<0.01)	
CK vs MNPK	**0.53**(p<0.01)	**0.43**(p<0.01)	**0.34**(p<0.01)	
M vs NPK	**0.58**(p<0.01)	**0.47**(p<0.01)	**0.30**(p<0.01)	
M vs MNPK	**0.59**(p<0.01)	**0.40**(p<0.01)	**0.27**(p<0.01)	
NPK vs MNPK	**0.51**(p<0.01)	**0.46**(p<0.01)	**0.26**(p<0.01)	
**Section of *Nitrospira*-like NOB**				
NxrB	**Treatments**			
**Aggregate size**	CK	M	NPK	MNPK
LA vs MA	ns	ns	ns	ns
LA vs SA	ns	ns	ns	ns
MA vs SA	ns	ns	ns	ns
	**Aggregate sizes**			
**Treatment**	LA	MA	SA	
CK vs M	**0.85**(p<0.01)	**0.87**(p<0.01)	**0.44**(p=0.022)	
CK vs NPK	**0.84**(p<0.01)	**0.87**(p<0.01)	**0.42(**p=0.038)	
CK vs MNPK	**0.84**(p<0.01)	**0.85**(p<0.01)	**0.41**(p=0.045)	
M vs NPK	**0.65**(p<0.01)	**0.78**(p<0.01)	**0.40**(p=0.032)	
M vs MNPK	**0.61**(p<0.01)	**0.71**(p<0.01)	**0.42**(p=0.022)	
NPK vs MNPK	**0.55**(p<0.01)	**0.62**(p<0.01)	**0.34**(p=0.022)	

Abbreviations: LA, macroaggregates (2000-250μm); MA, microaggregates (250- 53μm); SA, silt+clay fraction (<53μm). vs, versus; ns, no significant.

Table S4 Soil *Nitrobacter*- and *Nitrospira*-like NOB community PerMANOVA values showing the significant that fertilization and soil aggregate fractions contributed to the variation in the sequencing data

Source	*Nitrobacter*-like NOB		*Nitrospira*-like NOB	
	F-value	*P*-value	*F*-value	*P*-value
Fertilization	**28.2**	**<0.001**	**37.84**	**<0.001**
Soil aggregates	1.35	0.212	0.786	0.574
Fertilization*Soil aggregates	0.91	0.588	1.590	0.100

Table S5 Monte Carlo permutation tests by using redundancy analysis (RDA) to show correlation between the composition of *Nitrobacter*- and *Nitrospira*-like NOB community and soil geochemical variables. The data in bold indicated the effect was significant.

	Nitrobacter-like NOB		*Nitrospira*-like NOB	
	F-value	*P*-value	*F*-value	*P*-value
TC	**4.09**	**0.002**	**5.63**	**0.002**
TN	**4.52**	**0.002**	**6.41**	**0.002**
SOC	**3.68**	**0.002**	**4.95**	**0.002**
TP	**5.17**	**0.002**	**7.00**	**0.002**
TK	1.54	0.068	1.52	0.067
AP	**3.92**	**0.002**	**4.30**	**0.002**
AK	**4.93**	**0.002**	**5.21**	**0.002**
NH_4^+	**2.46**	**0.004**	**2.57**	**0.006**
